# A Rotation Alar Fold Flap for Cosmetic Nasal Plane Reconstruction: Description of the Technique and Outcome in Three Dogs

**DOI:** 10.3390/vetsci10110647

**Published:** 2023-11-09

**Authors:** Rosario Vallefuoco, Kadi Ritson, Frances Taylor, Caroline Fina, Alba Maria Bello

**Affiliations:** 1 Surgical Department, Pride Veterinary Referrals, Derby DE248HX, UK; kadi.ritson@scarsdalevets.com; 2 Oncology Department, Pride Veterinary Referrals, Derby DE248HX, UK; frances.taylor@scarsdalevets.com; 3 Diagnostic Imaging Department, Pride Veterinary Referrals, Derby DE248HX, UK; caroline.fina@scarsdalevets.com; 4 Internal Medicine Department, Pride Veterinary Referrals, Derby DE248HX, UK; alba.bello@ivcevidensia.es

**Keywords:** nasal reconstruction, planectomy, reconstructive surgery, oncologic surgery, head and neck, small animals

## Abstract

**Simple Summary:**

Cosmetic and functional outcomes are of great concern for nasal planum reconstruction, and the wishes of the owner in this regard may limit the extent of the excision and risk non-curative intent surgery. In veterinary medicine, several surgical techniques have been described, depending on the type, location, and extent of the tumor. In this study, a rotation alar fold flap technique is described and used in a short clinical case series following a subtotal or partial planectomy for oncological surgical purposes. No intraoperative and minor self-limiting post-operative complications were recorded, and the cosmetic and functional outcomes were considered very satisfactory in all cases. The rotation alar fold flap offers a valuable, feasible, functional, and aesthetically satisfactory alternative surgical option for selected cases of localized tumors involving the central and ventral planum. This technique, among others, can be used safely to improve the quality of life and the long survival time of our pets.

**Abstract:**

Nasal planum reconstruction is a surgical challenge, and several surgical techniques have been described. The objective of this study was to describe the rotation alar fold flap technique and to report clinical outcomes in a short case series. The feasibility of the technique was first assessed in a canine cadaveric model. The rotation alar fold flap was obtained by a single sharp horizontal incision of the dorsolateral nasal cartilage, preserving the caudal mucosal attachment to the ventral nasal concha. The flap was then rotated ventro-medially for the reconstruction of the ventral aspect of the nasal planum unilaterally or bilaterally. The rotation alar fold flap technique was used following a subtotal or partial planectomy for excision of a squamous cell carcinoma or mast cell tumors in three dogs. No intraoperative complications were recorded. Superficial surgical site infection was reported in two cases and minor dehiscence was reported in one case. However, survival of the flap was not affected. The cosmetic and functional outcomes were considered very satisfactory in all cases. The rotation alar fold flap technique offers a safe, valuable, feasible, functional and aesthetically satisfactory alternative surgical option for selected cases of localized tumor involving the central and ventral planum.

## 1. Introduction

Neoplasms of the nasal planum are relatively rare in dogs [1]. Squamous cell carcinoma (SCC) is the most commonly reported tumor [2,3], although other tumor types have been described in this location such as lymphoma, fibrosarcoma, hemangioma, melanoma, mast cell tumor (MCT), and fibroma [1,4]. Surgical excision of tumors of the nasal planum is the treatment of choice for the majority of tumor types, and it can be curative in selected cases if excision is complete [5,6]. However, nasal planum reconstruction remains surgically challenging due to constraints of normal anatomy, impacting options for wide local excision [7], and due to the lack of free local tissue to reconstruct the original appearance of the nose [8]. In veterinary medicine several surgical nasal reconstructive techniques have been described: purse-string reconstruction with second intention healing [4,9,10], musculofascial island labial flap [11], direct mucocutaneous apposition [12], bilateral labial mucocutaneous rotation-advancement flaps [5,13], modified nasal rotational flap [8], lip-to-nose flap [14,15], and recently, modified full-thickness labial/buccal rotational flap [16]. The choice of the most appropriate technique is based on the type, location, size, and extent of the tissue excision. Post-operative aesthetic outcomes after facial surgery can negatively affect the owner’s decision to proceed because the altered cosmesis may be unacceptable [4,9,10,12,17]. Furthermore, planectomy, with or without premaxillectomy, may be unnecessary for localized tumors, such as SCC, for which minimum lateral margins of 5 mm and 1 fascial deep plan can be achieved [7,15,17].

Subtotal planectomy for a tumor located in the central part of the nose without the involvement of the alar folds can be considered [11]. The alar fold flaps technique for nose reconstruction has been described for those cases by Pavletic (2010) [11]. Briefly, after the complete resection of the rostral lips, philtrum, and nasal planum, the spared alar folds are rotated upward and the cut ends are sutured together, whilst the dorsal borders of the alar folds are sutured to the adjacent cutaneous borders. Finally, bilateral lip advancement flaps are used to reconstruct the rostral lip defect. This technique improves the cosmetic results and helps shelter the exposed nasal mucosa from irritation secondary to environmental exposure [11].

The objective of this study is two-fold: (a) to describe a rotation alar fold flap technique following subtotal or partial planectomy, by assessing its feasibility in a cadaveric model, and (b) to report the outcomes in three dogs.

## 2. Materials and Methods

### 2.1. Ex Vivo Study for Surgical Technique Description

The feasibility of the technique was assessed on a canine cadaver (Labrador Retriever), euthanized for reasons unrelated to this study, and for which the owners donated the body post-mortem for teaching and research. The cadaver was stored at −18 °C and then thawed to room temperature for the feasibility study. The dog was positioned in sternal recumbency and its head was secured to the table with waterproof tape over a vacuum positioning cushion to maintain the most appropriate position for the surgical procedure. A subtotal planectomy was performed by removing, with a sharp incision, the central planum including the septum and the ventral planum including the philtrum and both nasal sulci, spearing the dorsal planum and the alar cartilage.

**Figure 1:**
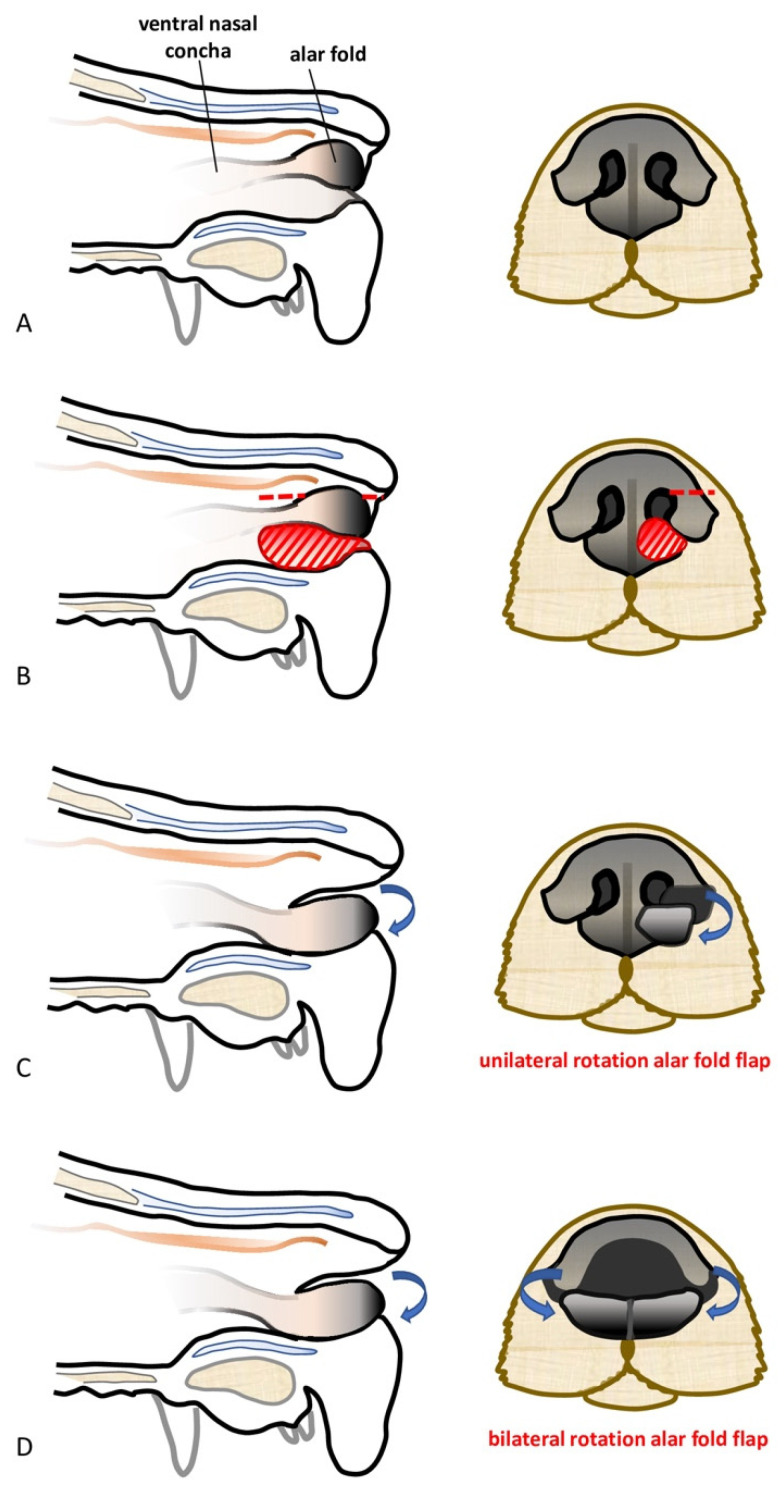
(**A**) The alar fold is an extension of the ventral nasal concha into the vestibule; (**B**) the red striped area represents the excised lower nasal planum and the red dotted line depicts the sharp incision of the left dorsolateral nasal cartilage with preservation of the caudolateral mucosal attachment to the ventral nasal concha; (**C**) the blue arrow represents the unilateral ventro-medial rotation of the left alar fold flap; and (**D**) bilateral rotation of the alar fold flaps.

**Figure 2:**
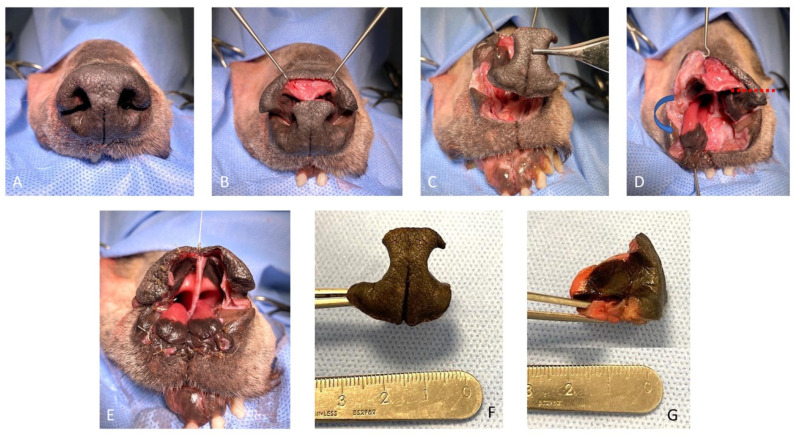
Surgical steps of the rotation alar flap technique following subtotal planectomy. (**A**) Pre-operative aspect of the nose; (**B**) sharp incision of the dorsal aspect of the central planum sparing the dorsal planum; (**C**) the septum and the central and ventral planum are removed, sparing the alar cartilages; (**D**) single sharp horizontal incision of the left dorsolateral nasal cartilage preserving the caudo-lateral mucosal attachment to the ventral nasal concha (red dotted line); the right alar flap is pivoted ventro-medially (blue arrow); (**E**) final aspect of the reconstructed nasal vestibulum and ventral aspect of the nasal planum; and (**F**,**G**) the excised septum and central and ventral planum.

The rotation alar fold flaps were obtained on both sides by a single sharp horizontal incision of the dorsolateral nasal cartilage, preserving the alar fold and its caudo-lateral mucosal attachment to the ventral nasal concha. The flaps were then pivoted ventro-medially for the reconstruction of the nasal vestibulum and ventral aspect of the nasal planum. The mucosal edges of the flap were sutured together in the middle and to the dorsal aspect of the rostral lips in a simple interrupted suture pattern with a 4.0 absorbable monofilament suture. The nasal septum and the central planum were not reconstructed (Figure 1 and Figure 2).

The feasibility of the same technique following unilateral partial planectomy was assessed in another canine cadaver (Labrador Retriever). Unilateral partial planectomy was performed, with the sulcus and ventral planum being unilaterally removed with a sharp incision, sparing the central planum, the philtrum, and the alar cartilage. The rotation alar fold flap was created in the same way described above and it was used to unilaterally reconstruct the nasal defect.

### 2.2. Case Reports

**Figure 3:**
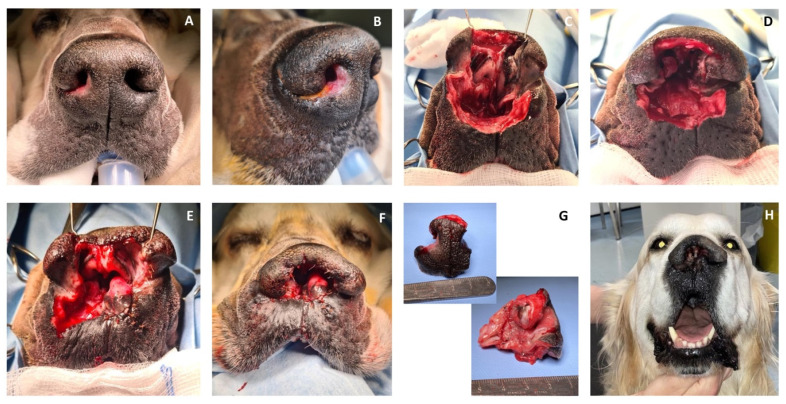
(**A**,**B**) Preoperative aspect of the nasal SCC; (**C**,**D**) resection of the septum and central and ventral planum, sparing the alar cartilages; (**E**,**F**) reconstruction of the nasal vestibulum and ventral aspect of the nasal planum; (**G**) the complete excised SCC; and (**H**) aspect of the dog 5 months after surgery.

*Case 1*. A 10-year-old, 36.5 kg, intact male Golden Retriever was referred for further investigations of a 4-month history of unilateral right serous nasal discharge associated with an ulcerative lesion involving the right rostral nostril. On admission, an ill-defined area of depigmented tissue within the medial margin of the right nare was visible. The submandibular and superficial cervical lymph nodes were palpably normal in size. Pre-operative complete blood count and serum biochemistry profiles were within normal range, and the serology for canine sino-nasal aspergillosis was negative. Rhinoscopy and computed tomography (CT) examination of the head, neck, and thorax were performed. Computed tomography of the nasal cavity revealed a poorly defined, contrast-enhancing soft-tissue attenuating mass involving the philtrum, the rostral, and the right lateral part of the septum. There was no evidence of bony destruction or thoracic abnormalities. Histopathology examination of the nasal biopsies was consistent with SCC. Cefalexin (20 mg/kg IV) was administered for antimicrobial prophylaxis at induction and every 90 min throughout the procedure. The patient was placed in sternal recumbency, and the nose and maxillary region were clipped and aseptically prepared. Two minutes before surgery, xylometazoline hydrochloride (0.1%) was sprayed into the nasal cavities with a nasal spray diffusor device. A subtotal planectomy was performed to achieve at least 5 mm gross surgical margins: the central planum including the septum and the ventral planum including the philtrum and both nasal sulci were excised with a sharp incision, sparing the dorsal planum and the alar cartilage. Nasal reconstruction was performed as described above for the ex vivo study (Figure 3).

The histopathology examination was consistent with complete excision of the SCC. Pain was managed in the hospital by methadone (0.2 mg/kg every 4 h, IV), meloxicam (0.1 mg/kg, SID, IV), and paracetamol (10 mg/kg, BID, IV). The dog was discharged 2 days postoperatively with a 7-day oral course of meloxicam (0.1 mg/kg), paracetamol (10 mg/kg, BID), and clavulanate amoxicillin (12.5 mg/kg BID). Upon discharge, the dog appeared comfortable and was eating and drinking normally. Clinical follow-up at 1 and 2 weeks after surgery showed progressive healing of the surgical wound without dehiscence. A moist superficial surgical site infection was present at 1 week post-op. The antibiotics course was continued for 1 more week. By the third week, the wound was completely healed. Owner satisfaction with the procedure and the appearance of the dog was very high. Clinical follow-up at 3 and 5 months was unremarkable: there were no signs of tumor recurrence or signs of any complications. Seven months after surgery, the owner reported that the dog was lethargic and inappetent. No visible signs of tumor recurrence were apparent or enlargement of the regional lymph nodes. Blood work abnormalities included severe increased ionized calcium (3.67 mmol/L- usual range 2.34–3.0 mmol/L). The owner declined further investigation and humane euthanasia was elected.

**Figure 4:**
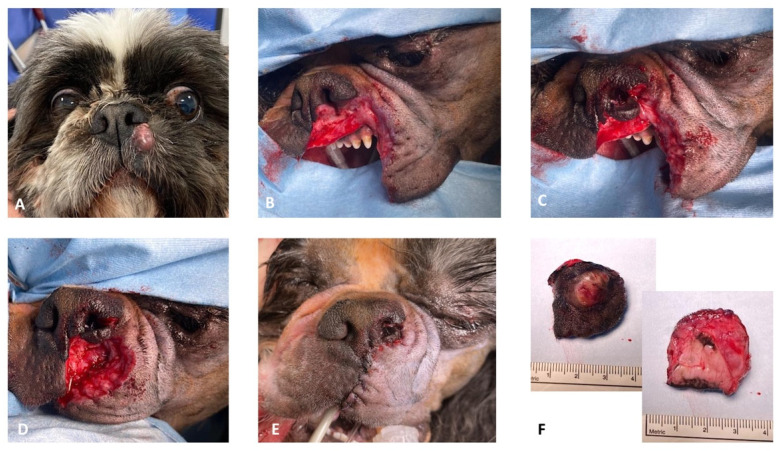
(**A**) Preoperative aspect of the left rostral lip MCT; (**B**) resection of the rostral lip and the left ventral planum; (**C**–**E**) consecutive steps for nasal and labial defect reconstruction; and (**F**) the complete excised MCT.

*Case 2*. A 12-year-old, 10 kg, intact male Shih-Tzu was referred for recurrence of an incomplete resected low-grade (Kiupel grading system) MCT affecting the left rostral lip. Physical examination revealed an approximately 1 cm round, alopecic cutaneous mass immediately ventro-lateral to the philtrum and the left ventral planum. The pre-operative complete blood count and serum biochemistry profiles were largely unremarkable. Abdominal ultrasound followed by ultrasound-guided hepatic and splenic fine-needle aspirates and cytology examination revealed no evidence of distant tumor metastasis. Sentinel lymph node mapping using a peri-tumoral intradermal injection of lipid-based contrast medium (Lipiodol Ultra-Fluid™), as previously described by Brissot et al. (2017) [18], revealed uptake of contrast in the left submandibular lymph node. Cefalexin (20 mg/kg IV) was administered for antimicrobial prophylaxis at induction and every 90 min throughout the procedure. Chlorphenamine maleate (0.5 mg/kg IM) was also administrated at induction. The patient was placed in right lateral recumbency with the head slightly elevated. Two minutes before surgery, xylometazoline hydrochloride (0.1%) was sprayed into the nasal cavities with a nasal spray diffusor device. After lymphadenectomy of the sentinel lymph node, MCT excision was performed with the largest possible proportional margins. The oral mucosa was considered the deep surgical margin. Dorsally, the surgical excision included the left ventral planum. The reconstruction of the rostral labial and nasal defects was achieved by a combined full-thickness labial advancement flap and a unilateral left side rotation alar fold flap as described above (Figure 4).

Histopathology examination of the excised tissue confirmed the complete excision of a high-grade (Patnaik and Kiupel grading system) MCT with no evidence of metastasis of the sentinel lymph node.

The dog was discharged the day after surgery, with a 7-day course of meloxicam (0.1 mg/kg) and clavulanate amoxicillin (12.5 mg/kg BID). Clinical follow-up 2 weeks after surgery revealed an uneventful healing process. According to the owners, the functional and aesthetic outcomes were very satisfactory. Due to financial constraints, no adjuvant treatment was planned for this dog. At long-term telephonic follow-up (26 months after surgery), the dog was still alive without visible signs of tumor recurrence.

**Figure 5:**
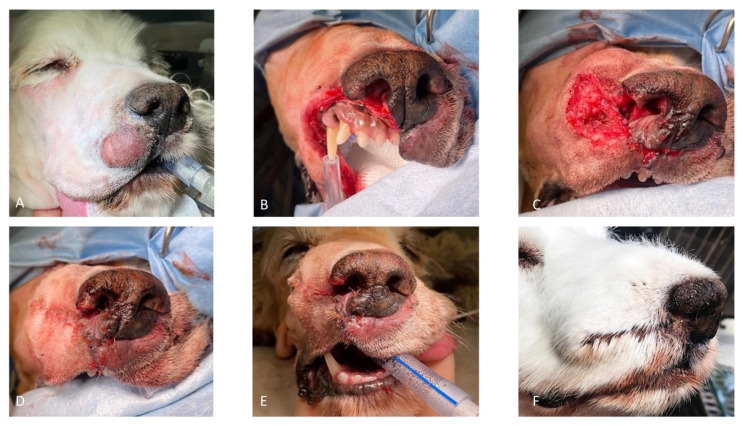
(**A**) Preoperative aspect of the right rostral lip MCT; (**B**) resection of the rostral lip and the right ventral planum; (**C**) combined unilateral rotation of the alar fold flap and full-thickness labial advancement flap for reconstruction of the nasal and labial defects; (**D**,**E**) final aspect of the lip and the nose; and (**F**) aspect of the dog 1 month after the surgery.

*Case 3*. A 12.5-year-old, 12.6 kg, intact male Cocker Spaniel was referred for unilateral right nasal discharge and a 3 cm cutaneous mass in the region of the right upper lip and rostral region. Cytology of the FNA was consistent with an MCT. The pre-operative complete blood count and serum biochemistry profiles were largely unremarkable. A CT scan of the head, neck, and thorax revealed a focal homogenous, soft tissue attenuating, contrast-enhancing mass in the right upper lip, whereas CT of the thorax was within normal limits with no overt signs of thoracic metastasis. CT-indirect lymphography using an intra-tumor injection of water-soluble contrast medium iohexol (Omnipaque™) revealed intense uptake of contrast within the right lateral mandibular lymph node and mild uptake of the right medial retropharyngeal lymph node. Abdominal ultrasound examination followed by ultrasound-guided hepatic and splenic fine needle aspiration and cytology examination revealed no evidence of tumor metastasis. Cefalexin (20 mg/kg IV) was administered for antimicrobial prophylaxis at induction and every 90 min throughout the procedure. Chlorphenamine maleate (0.5 mg/kg IM) was also administrated at induction. The patient was placed in left lateral recumbency with the head slightly elevated and at an approximately 45° angle when viewed from the front. Two minutes before surgery, xylometazoline hydrochloride (0.1%) was sprayed into the nasal cavities with a nasal spray diffusor device. Following lymphadenectomy of the right submandibular and right retropharyngeal lymph nodes, the tumor excision was performed with the largest possible proportional margins. The oral mucosa was considered the deep surgical margin. Dorsally, the surgical excision included the right lower planum. The reconstruction of the rostral labial and nasal defects was achieved by a combined full-thickness labial advancement flap and a unilateral right side rotation alar fold flap as described above (Figure 5).

The dog was discharged the day after surgery with a 7-day course of meloxicam (0.1 mg/kg SID) and clavulanate amoxicillin (12.5 mg/kg BID). The antibiotic course was protracted due to a local superficial surgical site infection and minor wound dehiscence of the tip of the labial flap.

Second-intention healing allowed the complete closure of the wound within 3 weeks of surgery. Owner satisfaction with the procedure and the appearance of the dog was very high. Histopathology examination was consistent with incomplete excision of a low-grade (Kiupel grading system) and high-grade (Patnaik grading system) MCT. Histopathological examination of the sentinel lymph nodes did not reveal evidence of tumor metastasis. Adjuvant chemotherapy was started 4 weeks after surgery. The protocol was based on vinblastine (2 mg/m^2^, IV, every week with 4 doses and then every 2 weeks with 4 further doses) and prednisolone (1 mg/kg, PO, SID, every day for 1 week, followed by 0.5 mg/kg SID, every other day) administration [19]. At clinical follow-up 4 months after the surgery, no local recurrence or nasal stricture were reported. At long-term telephonic follow-up (22 months after the surgery), the dog was still alive. The owner reported the growth of a 0.5 mm lesion at the level of the surgical wound, but they declined further investigations and treatment.

## 3. Discussion

The rotation alar fold flap technique described here was used successfully in three dogs for nasal reconstruction following subtotal and partial planectomy. No intraoperative complications directly associated with the surgical technique were observed and the post-operative complications were limited to local infection and limited wound dehiscence that did not affect the viability of the flap itself. This technique presents several advantages. It is relatively easy and safe to perform. Comparatively with complex skin flaps, the use of local planum tissue in the flap requires a limited amount of time, reducing the anesthesia time, the anesthetic risks related to a prolonged surgery as well as the cost [8]. The alar fold flap is a local rotation mucocutaneous flap that relies on an extensive interconnecting network of arteries and veins [11]. Furthermore, the rotation of the flap was limited up to 90°, reducing the risk of blood supply compromise. Nasal reconstruction using a local mucocutaneous flap maintained the mucocutaneous junction throughout and allowed single-stage primary wound healing with minimal tension, reducing the risk of scar tissue formation and consequently nostril stenosis [5]. From a functional point of view, in fact, the diameter of the nasal opening was preserved and maintained in the long term. Incorporating the alar fold into the flap allowed for the use of robust vascularized tissue with the same pigmented, non-haired appearance of the nose. In this way, the overall post-operative cosmetic appearance was not compromised, and it was considered satisfactory, according to the clients. In one dog (*case 1*), the philtrum and the septum were not reconstructed. However, that did not cause flattening of the dorsal planum and did not affect the final aesthetic or functional outcome. This confirms the satisfactory cosmetic outcome reported by Chiti et al. (2017) [14], where the dorsal planum was reconstructed by a lip-to-nose technique in one dog without reconstruction of the philtrum.

In the initial technique described by Pavletic (2010) [11], the alar fold flaps are bilaterally rotated upward to reconstruct the dorsal planum, whilst the lower planum and the rostral lip defect are reconstructed by bilateral lip advancement flaps. In our cases, this technique could not be used following the unilateral partial planectomy (*cases 2* and *3*) nor could it be used to reconstruct the large nasal vestibule defect after the subtotal planectomy (*case 1*). The rotation alar fold flap described here can be unilateral or bilateral based on the specific case needs, and it can also be used to reconstruct the nasal vestibule and the lower nasal planum due to its rotation in a ventro-medial direction. However, its use is limited to the reconstruction of the central and lower planum.

In our short case series, nasal reconstruction followed oncologic surgery. Obtaining surgical margins is a priority, and ideally, it should not be sacrificed at the expense of cosmetic outcomes [11]. However, indiscriminate removal of tissues in the absence of clinical judgment is also unsatisfactory, [11] in particular if the postoperative unacceptable results would prevent the owners from consenting to curative intent surgery [5]. Depending on the type, location, size, and tumor extent, it is possible to completely excise the tumor yet preserve portions of the nasal planum for reconstruction [11]. Margin intent surgery remains challenging, however, due to the constraints of anatomy and postoperative patient satisfactory cosmesis and functionality. In our short cases series, we aimed to achieve 5 mm margins for the SCC [15,17] involving the philtrum, the septum, and the ventral nasal planum of *case 1* and at least proportionate margins [20] for the MCT of *cases 2* and *3.* The excision was complete for *cases 1* and *2*, but it was incomplete for *case 3*. The MCT of *case 3* was graded as low grade according to the Kiupel grading system but high grade according to the Patnaik grading system, with no histological evidence of metastasis to the sentinel lymph node. Mast cell tumors occurring on the muzzle seem to behave more aggressively and are characterized by local infiltration [21]. However, the extent of the ideal surgical margin for an MCT in this location still remains unknown [7]. In our case, adjuvant chemotherapy following incomplete excision was planned and suspected local recurrence was noted on long-term follow-up.

Accurate preoperative evaluation of the extent of the tumor to define the margins of the excision is crucial [2,6]. Computed tomography and magnetic resonance imaging have proved to be valuable staging tools in dogs with tumors of the nostrils. They can help define the posterior extent of the tumor and guide the posterior level of resection or the posterior extent of the radiation field [1].

A contrast-enhanced computed tomography study, including the head, neck, and thorax, was performed in two of our cases, and surgical planning was based on the tomographic findings. Rhinoscopy was also performed in *case 1* to evaluate the extent of the lesion and also to rule out other nasal diseases. In *cases 2* and *3*, staging was completed with sentinel lymph node mapping using a peri-tumoral intradermal injection of contrast medium, abdominal ultrasound, and fine needle aspiration of the liver and spleen. In those two cases, the sentinel lymph nodes were excised too. Because local or distant metastatic disease from nasal planum SCC appears to be rare [2,3], in *case 1*, lymph node staging was limited to physical examination and diagnostic imaging. For this case, for which the excision of the tumor was complete, 7 months after surgery, hypercalcemia associated with severe clinical signs was diagnosed. The owner elected for humane euthanasia and declined further investigations and post-mortem necropsy. Hypercalcemia as a paraneoplastic syndrome of recurrent or metastatic SCC could have been possible; however, no signs of local recurrence or enlargement of regional lymph nodes were noticed at that time, and other pathologies or other types of tumor as a cause of hypercalcemia cannot be completely ruled out.

Intra- and post-operative bleeding is one of the major concerns during nasal and maxilla-facial surgeries. In order to reduce the rate and extent of bleeding and to improve the visibility of the surgical field, xylometazoline hydrochloride (0.1%) was sprayed into the nasal cavities in all of our cases. Xylometazoline is an imidazole derivative that acts directly on the alpha-adrenergic receptors of the arterioles of the nasal mucosa to produce vasoconstriction resulting in decreased blood flow. Its efficacy is comparable to the diluted adrenaline in the control of anterior epistaxis in human medicine [22]. The use of xylometazoline has also been suggested in veterinary medicine before rhinoplasty surgery in brachycephalic dogs [23].

Post-operative complications in maxilla-naso-facial surgery are common and frequently include incisional dehiscence or stenosis of the nasal opening, which is likely due to excessive tension [2,3,13,24], or chronic rhinitis and sneezing due to external exposure of the nasal mucosa to the environment [1].

Self-limiting superficial surgical infection occurred in two of our cases. This was the consequence of continuous licking of the wound. The Elizabethan collar, in fact, protected the wound from being scratched and rubbed with the legs and paws, but it was ineffective in protecting the wound from the tongue. The superficial infection did not affect the survival of the flap and no further management was required aside from a protracted course of antibiotics.

Minimal wound dehiscence was observed in *case 3*, where a combination of lip advancement-rotation alar flaps was performed. In this case, the dehiscence involved the tip of the labial flap. The tension and motion of this region play a major role in this complication. Also, in this case, no further treatment was required, aside from local care of the wound and a protracted antibiotic course.

## 4. Conclusions

In conclusion, we describe a rotation alar fold flap technique and its application in three clinical cases following subtotal or partial planectomy. This nasal reconstruction technique offers a safe, valuable, feasible, functional and aesthetically satisfactory surgical option. This technique could be used for selected cases of localized tumor involving the central and ventral nasal planum and for which the oncology surgical principle and the functional and cosmetic expectations of the client can be respected.

## Data Availability

No new data were created or analyzed in this study. Data sharing is not applicable to this article.
